# Primary Cilia: Highly Sophisticated Biological Sensors

**DOI:** 10.3390/s90907003

**Published:** 2009-09-03

**Authors:** Wissam A. Abou Alaiwi, Shao T. Lo, Surya M. Nauli

**Affiliations:** Pharmacology Department; MS 607 / University of Toledo, 2801 W. Bancroft Street, Toledo, OH 43606, USA; E-Mail: slo@utnet.utoledo.edu (S.T.L.)

**Keywords:** primary cilia, mechanosensory transduction, calcium, fluid shear stress

## Abstract

Primary cilia, thin hair-like structures protruding from the apical surface of most mammalian cells, have gained the attention of many researchers over the past decade. Primary cilia are microtubule-filled sensory organelles that are enclosed within the ciliary membrane. They originate at the cell surface from the mother centriole that becomes the mature basal body. In this review, we will discuss recent literatures on the roles of cilia as sophisticated sensory organelles. With particular emphasis on vascular endothelia and renal epithelia, the mechanosensory role of cilia in sensing fluid shear stress will be discussed. Also highlighted is the ciliary involvement in cell cycle regulation, development, cell signaling and cancer. Finally, primary cilia-related disorders will be briefly described.

## 1. Introduction

Despite their discovery over a century ago, primary non-motile cilia were thought to be vestigial organelles inherited from an ancestor whose cells had motile flagella, and that the flagella or cilia now served no purpose. In particular, although the presence of cilia has been observed in various cell types in mammalian cells [1,2], the function of cilia continued to elude researchers for many decades. Most mammalian cells posses a solitary, non-motile cilium known as primary cilium which projects from the apical surface of polarized and differentiated cells to the internal lumen of the tissues.

Historically, cilia have been studied for their motile function in fluid and cell movement [3]. Functions of motile cilia have been studied extensively in lung epithelial cells, sperm tails, and other systems. In addition, the building blocks of motile cilia have also been studied intensively in green algae [4]. Most recent works have further shifted to look at primary, non-motile cilia in mammalian systems [5]. Non-motile cilia have acquired much attention over the last few years, because ciliary defects contribute to various human diseases such as cystic kidney disease [6,7].

**Figure 1.**
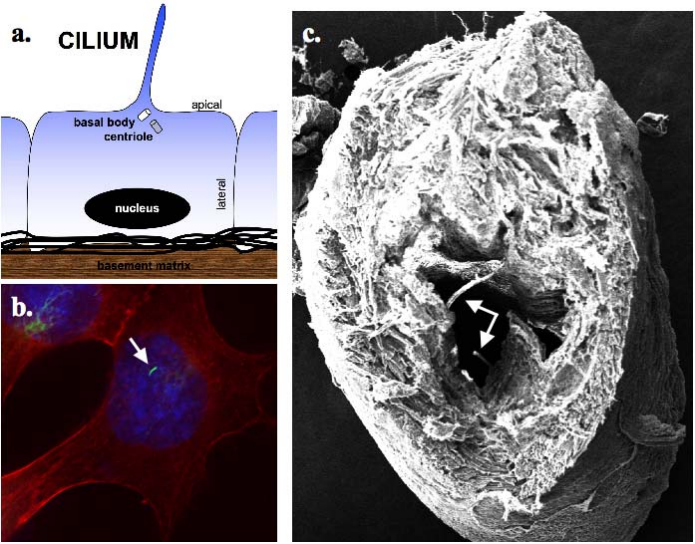
Cilia are sensory organelles that project from the apical sides of cells. **(a)** A cartoon depicts extension of cilia from one of the centrioles, which is termed basal body. **(b)** Immunostaing study shows the presence of a cilium of an endothelial cell. **(c)** Electron micrograph further confirms the presence of cilia in the lumen of embryonic aorta *in vivo*. Arrows indicate the presence of cilia.

Like the mitochondria, Golgi apparatus and the endoplasmic reticulum, cilia function as specialized cellular organelles. All cilia are formed during interphase of the cell cycle from an ancestral basal body or elder centriole of the centrosome [8]. The centrosome is composed of the two centrioles that nucleate the bipolar formation of the mitotic spindle during mitosis and nucleate the ciliary axoneme (Figure 1). The association between cilia and centrioles has opened a possibility for a ciliary role of in cell division. Disassembly of the cilia prior to mitosis has thus been hypothesized to be a “checkpoint” for cells to undergo division. Hence, disassembly of the primary cilia and the subsequent liberation of the centrioles are thought to be essential for cell division [2]. Whether or not disassembly of cilia would be a universal cell cycle checkpoint for all mammalian cells remains to be established in the future.

Recent advances have shown that cilia from protists to humans might carry out sensory functions and can be either motile or immotile. Most motile cilia (or flagella) consist of 9+2 axonemes assembled by nine peripheral microtubule doublets and two central single microtubules in addition to other associated structures such as an inner and outer dynein arms, radial spokes and nexin links. An example for this can be found in the *Chlamydomonas* flagellum, which is a motile organelle with 9+2 axoneme. On the other hand, most non-motile cilia consist of axonemes lacking the two central microtubules. An example of this includes endothelial cilium, which is a non-motile organelle with 9+0 axoneme. Although a diversity of cilia exist with many different classes and variation, all cilia types share basic structural units of microtubule doublets and ciliary membrane [9].

The presence of different cilia types is an indication that cilia have different functions. Among motile cilia, the axonemes with nine peripheral doublets and attached dynein arms have dynein heavy chains responsible for ciliary movement. This movement is ATP-dependent and happens through conformational changes and transient binding to nearby microtubule doublets. Cilia motility is generated through the coordinated activation and inactivation of the dynein motor proteins along the axoneme [10]. One example is the motility of the 9+2 cilia of the respiratory tract epithelium for mucociliary clearance [11]. On the other hand, functions of cilia that do not involve motility are implicated in sensing the environmental signals. Acting as biological sensors, cilia function as antenna that receive information from the surrounding environment and transduce the message through signaling cascades into the cell body. Hence, the ciliary membrane harbors many cilia-specific receptors, ion channels and sensory signaling molecules and complexes.

## 2. How Are Cilia Structured, and What Are Cilia Made of?

Primary cilia are filled with a microtubule-based cytoskeleton which forms the ciliary axoneme. The axonemal microtubule is arranged in doublets 9+0 organization pattern that fits with the pattern of the mother centriole. The mother centriole or basal body emanates a cilium and later develops into the basal body during G_0_ [5]. This axonemal extension is enclosed by the ciliary membrane, an extension of the cell membrane (Figure 1). Most of what is known today about the assembly of primary cilia came from studies of the biflagellated green alga *Chlamydomonas*. The experimental advantages of *Chlamydomonas* paved the way for the discovery and elucidation of the process by which cilia or flagella are assembled, known as intraflagellar transport (IFT) [4,12]. The IFT is a highly conserved mechanism among ciliated organisms from unicellular organisms to humans. As mentioned earlier, the assembly of cilia is tightly coupled to the cell cycle, and cilia emanates from the mother centriole as cells proceed to growth arrest or G_0_, whereas cilia are disassembled in cells shortly before entering mitosis [3]. According to Sorokin [8], ciliary assembly passes through three distinct stages. *First*, there is the appearance of a Golgi-derived vesicle that becomes attached to the distal end of the mother centriole from which the axoneme starts to emanate. This centriole later elongates and becomes the distal basal body. *Second*, the accumulation and fusion of nearby vesicles form the sheath around the axonemal shaft, which elongates through microtubule assembly. *Third*, the elongating axoneme surrounded by the ciliary-membrane fuses with the cell membrane and forms the ciliary necklace. Further assembly of the ciliary axoneme is mediated by IFT, an active bi-directional movement of motor protein between the outer axonemal segments and the ciliary membrane. The IFT proteins are moved from the base of the cilia to the distal ciliary tip by kinesin-2 motor proteins and reshuffled back to the cell cytoplasm by dynein-2 [4].

In general, cilia are made up of four main sub-ciliary compartments: 1) the membrane domain, a specialized domain composed of a protein and with a lipid composition different from that of the rest of the plasma membrane. Ciliary membrane houses the many sensory receptors and channels to support sensory functions of cilia; 2) *the soluble compartment*, also known as the matrix compartment or cilioplasm. The matrix constitutes of the fluid material between the ciliary membrane and the axoneme where the IFT machinery is located to assemble and maintain the cilia or flagella; 3) *the axoneme*, a tubulin-based structure that plays essential roles for motor proteins to transport ciliary components to the cilia. Axoneme is composed of 9 pairs of microtubules, which are posttranslational modified to support the long ciliary structure; 4) *the tip*, the distal part of the axoneme. Although the role of ciliary tip is still unknown, the ciliary tip contains protein complexes that have been proposed to have specialized functions [13]. The overall protein composition of cilia is very complex, considering the function and structure of cilia. It has been estimated that cilia contain over 1,000 different proteins [14–17]. Thus, the most daunting tasks are to confirm the functions of these proteins.

## 3. Primary Cilia and Sensing

**Figure 2.**
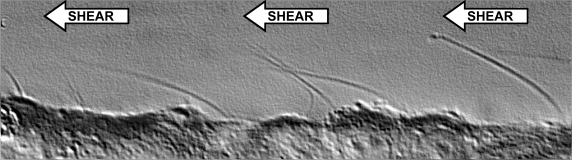
Cilia are sensory organelles that sense fluid-shear stress on the apical membrane of the cells. Fluid flow that produces enough drag-force on the top of the cells will bend sensory cilia. This biomechanical properties play a very important role in vestibular organs that support bodily fluid perfusion.

It was only recently that physiologists paid considerable attention to studying the mechanical properties of primary cilia when Schwartz *et al*. proposed that primary cilia of renal cells grown in culture might have a flow sensory function [18]. This hypothesis was based on observations that primary cilia in culture can bend considerably in response to fluid flow when subjected to flow rates (Figure 2). The hypothesis of fluid mechano-sensing by the cilia was later investigated and confirmed by Praetorius and Spring [19]. They demonstrated that primary cilia of Madin-Darby canine kidney (MCDK) cells were able to respond to fluid and mechanical stress, induced by increasing the fluid flow rate or cilia bending with a micropipette. The mechanism of fluid flow sensing and ciliary-related sensory proteins were further confirmed and identified [20,21]. Cilia are composed of polycystin-1 and polycystin-2, which function as mechano-sensor complex. Polycystin-2 has been shown to act as a calcium channel. Blocking polycystin-1 function will inhibit the polycystin-2-mediated intracellular calcium mobilization.

The mechanosensory polycystins have since been studied in primary cilia of other cell types. The mechanosensory functions of primary cilia and polycystins have been further described in the kidney epithelia [22–24], osteochondrocytes [25,26], cholangiocytes [27] and developing nodes [28]. Most recently, it was shown that similar to primary cilia from other systems, cilia from endothelial cells isolated from embryonic mouse aortas are able to respond to fluid flow shear stress that mimic the blood flow in blood vessels by a similar mechanism and induce an increase in intracellular calcium that is dependent on the structural polaris molecule and the mechanosensing polycystin-1 [29]. It was demonstrated that primary cilia play critical and distinct roles in sensing and transducing extracellular signals such as fluid shear stress into intracellular biochemical responses such as calcium signaling and nitric oxide synthesis. Nauli’s laboratory further demonstrates that polycystin-2 is localized to cilia of mouse and human endothelial cells and is required for endothelial cilia to sense fluid shear stress [30]. It was proposed that ciliary polycystin-2 is a shear-sensitive calcium channel required to activate a biochemical cascade for nitric oxide (NO) production. The mechanism of fluid shear sensing is mediated through a complex biochemical cascade that involves calcium, calmodulin, Akt/PKB and protein kinase C and leads to the synthesis of NO. Polycystin-2 functions as a mechanosensory calcium channel in endothelial cells and is involved in NO production, which in turn participates in controlling vascular tone and systemic blood pressure [30].

The role of cilia as sensory organelles is also demonstrated in olfactory and photoreceptor signaling. The binding of the olfactory ligands to their specific olfactory receptors localized to the ciliary membrane of olfactory sensory neurons leads to the activation of the olfactory signaling cascade through the production of the second messenger cyclic adenosine monophosphate (cAMP) within the cilium, leading to the depolarization of the cell by opening cyclic nucleotide gated channel that is located in the cilia [31,32]. Hence, cilia-less olfactory neurons lack odorant sensation. In a similar fashion to olfactory sensation, photoreceptor is mediated through the primary cilium in the cone and rod cells of the retina. This cilium is characterized by a specialized tip called the outer segment where the photo receptors that receive and initiate the reception of light are localized and the signal is initiated by an increase in cyclic guanosine monophosphate (cGMP) instead of cAMP, hence causing the closure of cGMP channels [33]. Maintenance of this signaling cascade requires the transport of retinal proteins such as Rhodopsin to the cilium through the IFT-mediated transport. Mutations that inhibit the transport of this protein cause a form of retinal degeneration called retinitis pigmentosa [34]. The role of cilia in sensing of smell and light is best manifested in Bardet-Biedl syndrome [35], a disease with non-functional cilia and basal body in which the patients suffer from lack of smell, retinal degeneration and the development of numerous complications such as polycystic kidney disease, obesity, polydactyly, hearing loss and others, emphasizing the important role primary cilia play in human health and disease [36–38].

Another sensory function associated with cilia is movement sensing. Movement sensing is mediated through the localization of members of the transient receptor potential (TRP) ion channels to the cilia of auditory and sensory neurons of *Drosophila* and *C. elegans* in which they respond to sound vibrations, nose touch and high osmolarity [39,40].

The function of primary cilia as cellular sensors for the skeleton has been previously shown [41] and recently reviewed [42]. Primary cilia have been described to have a similar mechano-sensory function in bone cells as in renal cells by which they can sense oscillatory fluid flow generated by mechanical stimuli during dynamic loading [43,44]. In addition to their role as mechanosensors in bone cells, cilia can also function as chemosensors through the localization of specialized receptors to their membrane that receives chemical signals from the extracellular environment and transmits it intracellularly. An example of well-studied chemosensory function of cilia is the Hedgehog signaling pathway. The Hedgehog signaling pathway is mediated through the Hedgehog complex composed of two trans-membrane proteins, Smoothened and Patched, that function at the primary cilium. The presence of Patched as a receptor at the primary cilium is to sense and bind to Shh in the environment and later leave the cilium for Smoothened [45]. The signaling pathway of Hedgehog in the cilia has been elaborated by many laboratories [13,46,47].

Interestingly, cilia have also recently been proposed to function as transducers of gravitational force as well as regulators of transcriptional noise. Moorman and Shorr [48] demonstrated that a change in gravitational force can be linked to variability in gene expression in rohon beard neurons of the zebrafish embryo, and this variability is mediated through the presence of primary cilium. This was further confirmed after selectively destroying the primary cilia in these neurons with chloral hydrate, resulting in homogeneity in gene expression of neuronal cells subjected to different gravitational forces.

## 4. Cilia and Cell Cycle Regulation

In most cells, there is an interesting association between ciliogenesis and cell cycle progression that is highly regulated. Most cells are ciliated in the stationary phase, or G_0_, of the cell cycle. In most cases, entry into mitosis requires ciliary disassembly [2,3]. One would expect that if cilia mediate signaling mechanisms that help the cells maintain their differentiated states or enter into mitosis, then ciliary abnormalities would lead to disruption of the balance between cell proliferation and cell death. Hence, it would cause proliferative disorders such as cancer, cystic kidney disease, cystic fibrosis and others. A growing body of evidence ranks cilia as the major contributor to cyst development in patients with cystic kidney disease.

A wide range of ciliary proteins have been indicated to play an important role in cell cycle regulation [49,50]. Many laboratories have now independently shown that ciliary polycystin-1 [20,24,27], polycystin-2 [27,28,30], polaris [29,51,52] and fibrocystin [53,54] play a mechano-sensory role through an increase in intracellular calcium concentration. The question becomes whether and how the ciliary proteins are involved in regulating cell cycle progression. One proposed mechanism by which polycystin complex might regulate cellular differentiation is by activating the cyclin-dependent kinase p21 via JAK-STAT and inhibition of proliferation induced by cAMP through Akt [55,56]. Recently, Aguiari *et al*. [57] showed that depletion of endogenous polycystin-1 leads to an increase in serum-induced calcium oscillations and cell cycle progression, providing a molecular link between calcium homeostasis and cellular proliferation through a ciliary mechanism. Moreover, the role of polycystins in abnormal cell proliferation has been demonstrated in two separate studies. Battini *et al*. [58] reported that loss of polycystin-1 function was associated with centrosome amplification and multiple mitotic abnormalities leading to genomic instability and polyploidism. Similar phenomena have been observed in polycystin-2 transgenic animal models in which polycystin-2 overexpression in mouse models was associated with chromosomal abnormalities, mitotic instability and centrosome overduplication [59].

Members of the NimA-related Kinase (Nek) family are important cell cycle kinases that are also localized to cilia. In particular, Nek Fa2p plays a role in the G2-M transition and is important for flagella disassembly in *Chlamydomonas* [60]. Moreover, Nek Cnk2p controls flagella length and cell size control prior to mitotic entry [61]. Recently, White and Quarmby [62] proposed that Nek1 also participates in the regulated coordination between ciliogenesis and cell cycle progression due to its role in centrosome integrity, which affects both ciliogenesis and centrosome stability.

In particular, studies in *Chlamydomonas* has revealed that removal of the IFT proteins, which are involved in protein trafficking in the cilia, has led to cell cycle or growth abnormalities. For example, low levels of IFT-27 led to cell growth retardation and asymmetric cell division [63]. In addition, IFT-88 overexpression prevented G1-S transition, and its inhibition resulted in ciliary disassembly and cell cycle progression [64].

All in all, it is clear that there is an intriguing relationship between the cilia, the centrosome and the cell cycle. The exact players in this link, however, are not yet clearly revealed. Nonetheless, it is expected that this area of research will lead to the discovery of new key players in the regulation of cellular proliferation, differentiation and cell cycle progression.

## 5. Cilia and Development

In addition to their localization to adult tissue, cilia are also found to be important regulators during embryonic development [6]. The idea that many diseases caused by ciliary defects have developmental manifestations prompted scientists to explore how ciliary function/dysfunction might contribute to developmental pathways.

By far, the most studied developmental pathway is the Hedgehog (Hh) signaling pathway due to its critical roles in the development of left-right asymmetry, neural tube closure and patterning, in addition to limb formation and others [65]. Evidence for cilia association with Hh signaling first came from mouse models with IFT or ciliary defects that exhibit characteristics of developmental abnormalities similar to abnormal Hh signaling. Such abnormalities include randomized left-right axis specifications, neural tube patterning defects and polydactyly [47,66–68]. Further studies show that many protein machineries involved in the Hh signaling are localized to and depend on primary cilia. Cilia, with their protrusion into the extracellular environment for ligand reception, become specialized organelles that are uniquely suited for this functionality.

In addition, the involvement of primary cilia in development regulation has gathered a growing interest in recent years as researchers study the role that primary cilia might play in planar cell polarity (PCP). PCP is the organized arrangement of cells in a plane of tissue perpendicular to the apical-basal axis through an oriented cell division. This organized arrangement is mainly mediated by a group of PCP core proteins including *frizzled* (*fz*), *disheveled* (*dsh*), *prickle* (*pk*), *diego* (*dgo*) and others [69–72]. A strong link between cilia function and PCP came from studies on different mouse models of cystic kidney disease designed to test whether cystogenesis might result from defective cell division orientation [73–76]. Results from these studies lead to the hypothesis that a correct orientation during cell division is required for the proper elongation of thin kidney tubules. Further evidence for ciliary involvement in PCP came from studies on inversin. Inversin is a ciliary-associated protein that regulates the balance between the canonical signaling and non-canonical (PCP) Wnt signaling [77]. All in all, a growing body of evidence has implicated structurally- and/or functionally-defective primary cilia in cystic kidney diseases and other disorders [78].

## 6. Cilia in Renal and Cardiovascular Disease

Primary cilia of renal epithelial cells are mechano-sensory organelles that can sense and respond to fluid flow by bending, resulting in an increase in intracellular Ca^2+^ concentration [19]. This response, when inhibited through an IFT (*Tg737/IFT88*) mutation, results in the development of cystic kidney [79]. In the kidney, the signaling molecules responsible for transmitting and articulating this flow sensing response are polycystin complex. This complex along the ciliary membrane is activated upon bending of the primary cilium, causing the influx of intracellular calcium and triggering activation of effector signaling cascades [3,36]. This sensory and signaling function of primary cilia involving polycystins has recently been demonstrated in mouse and human aortic endothelial cells [29,30]. Nauli *et al*. [29] demonstrated that immortalized endothelial cells have a single primary cilium responsible for fluid shear sensing and that proper localization and function of the mechanosensitive polycystin-1 within the cilia is crucial for proper communication with the extracellular environment. It was further shown that endothelial cells with *Pkd1^null/null^* or *Tg737^orpk/orpk^* mutation encoding for polycystin-1 or polaris, respectively, fail to transmit extracellular shear stress signals into intracellular calcium release and NO biosynthesis. Moreover, basal body-associated polcystin-1 in *Tg737^orpk/orpk^* endothelial cells is not sufficient to induce a fluid shear response.

**Figure 3.**
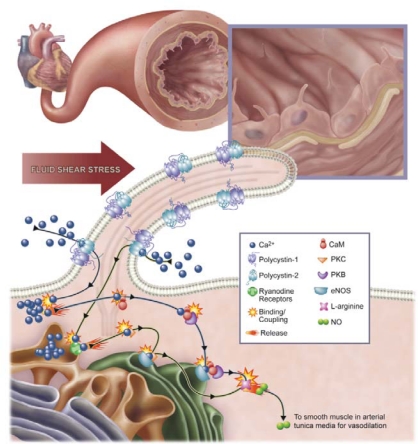
The presence of cilia in vasculature plays an important role in the biochemical production of a potent vasodilator, nitric oxide (NO). The figure depicts production of NO in an artery. Increases in blood pressure, which is translated to higher vascular shear stress, will be sensed by mechanosensory cilia. Bending or activation of the cilia involves mechanosensory polycystin-1 and polycystin-2 complex and a cascade of biochemical synthesis of NO. The cascade will further involve extracellular calcium influx (Ca^2+^), followed by activation of various calcium-dependent proteins, including calmodulin (CaM) and protein kinase C (PKC). Together with PKB, CaM and PKC are important downstream molecular components to activate endothelial nitric oxide synthase (eNOS).

Most recently, AbouAlaiwi *et al*. also showed that polycystin-2 is localized to the primary cilia of endothelial cells and functions as a calcium channel [30]. This localization is necessary for its sensory function, and it depends on the presence of a functional polycystin-1. This was demonstrated in cilia of both human and mouse cells. Abnormal polycystin-2 function or expression has been associated with the development of hypertension [80], probably due to the inability of endothelial cells to translate the increase in mechanical blood flow into cellular nitric oxide (NO), which controls vascular tone and blood pressure. Ciliary polycystin-2 might thus contribute to blood pressure maintenance through production of NO in endothelial cells. The mechanism involves complex biochemical components, including calcium, calmodulin, Akt/PKB, and protein kinase C (Figure 3).

The role of primary cilia as a fluid shear stress mechanosensor in the cardiovascular system has also been studied extensively by Poelmann, Hierck and colleagues [81–84]. The distribution of ciliated endothelial cells in the embryonic cardiovascular system is believed to be shear-related. Moreover, they show that there is a link between the localization and frequency of cilia in endothelial cells and the development of atherosclerosis. This was demonstrated through immunohistochemical localization analyses in wild-type as well as apolipoprotein-E-deficient mice. In particular, apolipoprotein-E-deficient mice are characterized by the development of spontaneous atherosclerotic regions in which significantly higher numbers of primary cilia were distributed around the atherosclerotic regions [84]. In addition, cultured ciliated endothelial cells from the embryonic heart and non-ciliated cells from the arteries demonstrated that when subjected to fluid shear stress, non-ciliated cells showed less induction of the shear marker *Kruppel-Like Factor-2* (*KLF2*) compared to ciliated cells. They also provided evidence that microtubule integrity is essential for cilia-related shear stress sensing. This prompted them to dissect the shear stress sensing mechanism into two steps. First, they noted *an immediate response*, which involves ciliary bending, polycystin activation, an increase in intracellular calcium, and a subsequent release of vasoactive substances such as NO. Following the immediate response was *a prolonged, long-lasting response* mediated through cytoskeletal rearrangements and transcriptional activation of shear-specific marker genes such as *KLF2*, which control cellular remodeling and phenotypic adaptation [82].

## 7. Cilia Involvement in Cell Signaling and Cancer

Another example of ciliary involvement in cell signaling is the involvement of primary cilia in the platelet-derived growth factor (PDGF) signaling pathway which contributes to normal embryogenesis and inflammation and is upregulated during carcinogenesis. The study by Schneider *et al*. [85] demonstrates that PDGFRαα as well as an effector downstream molecule (Mek1/2), but not PDGFRβ, preferentially localize to primary cilia of primary mouse embryonic fibroblasts and growth-arrested NIH/3T3 fibroblasts. The same study indicates that PDGF-AA stimulation of these fibroblasts resulted in PDGFRαα receptor phosphorylation and activation of the Akt and Mek1/2-Erk1/2 pathways. This response was absent from fibroblasts isolated from the cilialess mutant mice, thus confirming a coordination between ciliary assembly/disassembly and PDGF signaling pathway. In particular, mutations in the PDGFRα are implicated in multiple human malignancies, including gliomas and gastrointestinal stromal tumors [86,87]. Studying the perturbation of the PDGF signaling pathway through the primary cilia is thus important for understanding this malignancy and provides novel insights into its pathophysiology.

An additional area of interest that links cilia to cell signaling and cancer is concentrated on the cell cycle regulatory serine/threonine kinase, Aurora A. Aurora A localizes to the centrosome and coordinates bipolar spindle formation and nuclear envelope breakdown during mitosis, and it is overexpressed in certain tumors [88,89]. HEF1, a prometastatic factor overexpressed in certain melanomas and glioblastomas and found to have a centrosomal pool, can bind and activate Aurora A. HEF1 overexpression produces mitotic defects similar to those produced by Aurora A overexpression, suggesting a link between the two proteins functions [90]. It has been shown recently that Aurora A activity is necessary and sufficient to induce cilium resorption, probably through phosphorylation of the tubulin deacetylase HDAC6 [91].

Cancer cells have been proposed to have altered or reduced response to extracellular environmental factors because they lack primary cilia where most critical signaling molecules and receptor are localized [13,85]. Besides the ciliary involvement in cell signaling and/or cancer through the polycystins complex, the PDGFα, the Hedgehog, the Wnt, and the PCP pathways, other pathways that link cilia to cancer development exist, such as the von Hippel-Lindau and glycogen synthase kinase 3 β (VHL/GSK3β) pathways, in which the ability of cells to form cilia has been hindered through dysregulated signaling proteins expression [92]. Further investigations focusing on elucidating the relationship between the cilium, cell-signaling and cancer would definitely help researchers understand many of the pathophysiological mechanisms of different malignancies and target important ciliary proteins for therapeutic interventions.

## 8. Ciliopathies

Cilia are located on most cell types of the human body; hence ciliopathies or ciliary defects can affect single organs or have a multisystemic nature with clinically distinct and overlapping phenotypes [7,93]. In this section, primary cilia-related disorders will be briefly mentioned.

The kidney, liver and pancreas are characterized by tubular systems that transport fluid in the form of urine, bile or pancreatic secretions, respectively. Primary cilia protrude into the lumen of these functional tubular units as a fluid mechano-sensor. The development of cysts in the kidney, liver and pancreas in patients with autosomal dominant polycystic kidney disease (ADPKD) and nephronophthisis (NPHP) suggests that cystogenesis in these organs might involve a common mechanism. This mechanism has been proposed to be cilia-related simply because proteins involved in Bardet-Biedl syndrome (BBS), NPHP, orofacial digital syndrome type1 (OFD1), autosomal recessive polycystic kidney disease (ARPKD), and ADPKD localize to the cilium [94].

In a similar fashion to renal cilia [95], cilia localized to cholangiocytes of the bile duct can detect and transmit fluid flow signals to the epithelial cells and induce an intracellular calcium release response [96]. The fluid flow sensing might therefore provide a morphogenic stimulus that controls tubule diameter, which when lost can lead to abnormal growth control and cystogenesis.

Another abnormal sensory function of the cilia is manifested in retinal degeneration, in which mutations in *retinitis pigmentosa guanosine triphosphatase (GTPase) regulator* (*RPGR*) that is localized to the cilia contribute to 20% of retinitis pigmentosa cases [97]. In retinal rod and cone cells, all the components necessary for the assembly and maintenance of the outer segment are made in the cytosol and transported to the cilium by IFT. Disruption of IFT transport leads to retinal degeneration which can be associated with NPHP, BBS and Alstrom syndrome (ALMS). All of these pathologies share a common disrupted IFT or ciliary behavior [98–100].

Olfactory sensory neurons respond to olfactory stimulus by binding odorant ligands to specialized olfactory receptors located on the membrane of olfactory sensory cilia. This binding triggers a calcium signal which is later translated into active potential by the sensory neurons. A link between ciliary function and smell came from observations of patients with BBS and mice models with defective BBS genes who were unable to smell [101]. Similarly, patients with Usher syndrome (USH) suffer from a sensorineural hearing defect in which the USH proteins involved are localized or function in the stereocilia [102].

## 9. Future Prospectives

Although cilia are tiny micro-sensory organelles, they are highly complex, specialized structures involved in numerous functions. Hence, their dysfunction can lead to numerous disorders. Recent studies linking cilia function to the regulation of cell division and apoptosis might contribute to the advancement in understanding the mechanisms involved in carcinogenesis and help in the discovery of novel targets for therapeutic intervention in human cancer and other disorders such as cystic kidney disease. A better understanding of the cilia-related disorders as well as of the composition and functional role of cilia-associated proteins will provide novel insights into the sensory function of primary cilia.
